# Supplementary figures and images for: Membrane potential dynamics of excitatory and inhibitory neurons in mouse barrel cortex during active whisker sensing
Source: PLoS One. 2023 Jun 13;18(6):e0287174. doi: 10.1371/journal.pone.0287174 (PMC10263341; doi:10.1371/journal.pone.0287174)

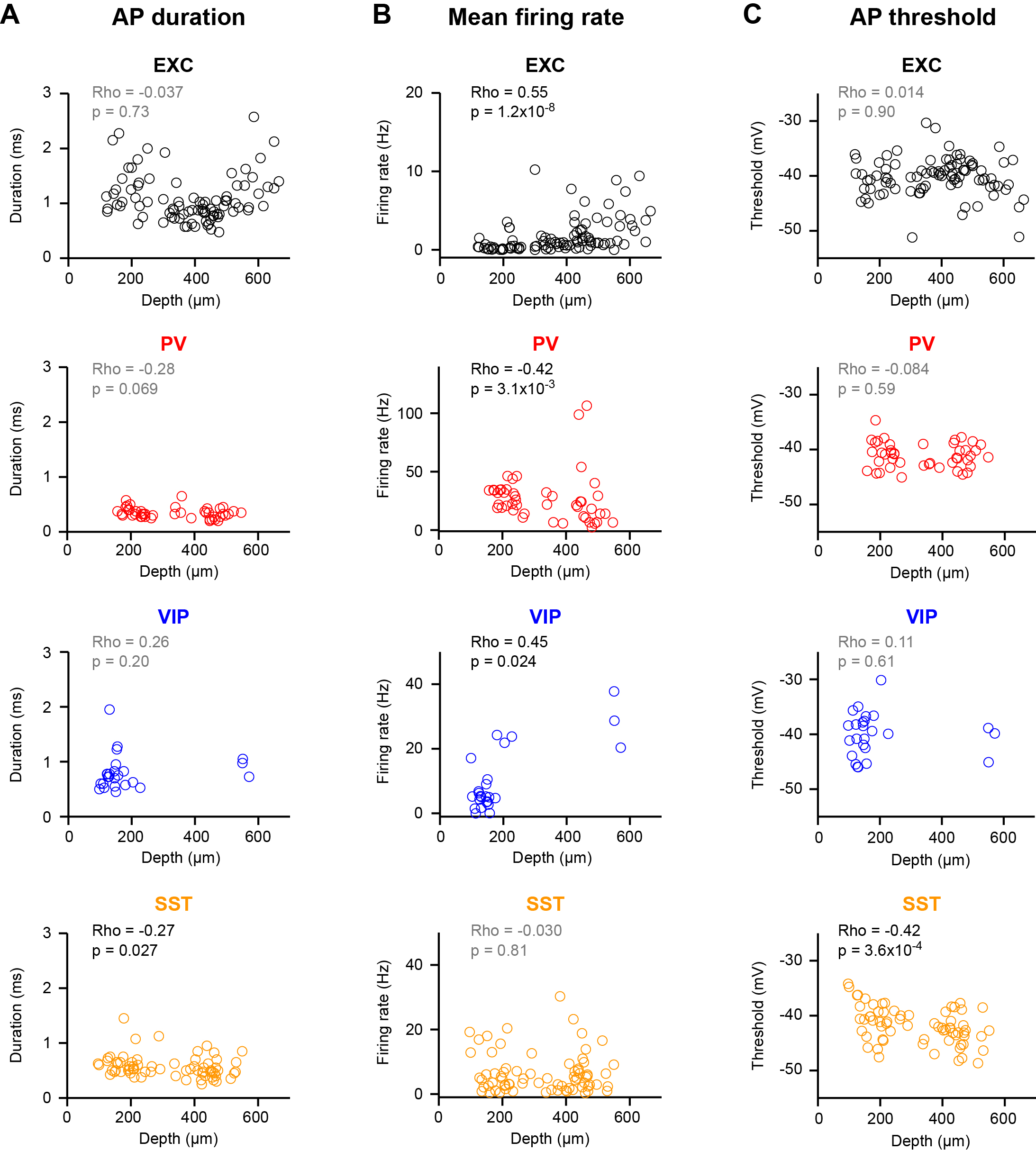

Supplement: S1 Fig — Related to Fig 2. (A) Mean action potential (AP) duration for epochs of quiet wakefulness across cell depth for each cell class. Open circles represent single neurons. Correlation between firing rate and cell depth was assessed using a Spearman test; Spearman correlation coefficient (Rho) and p value are indicated on each graph. (B) Same as A, but for the mean AP firing rate. (C) Same as A, but for the mean AP threshold. (JPG) [file pone.0287174.s005.jpg]

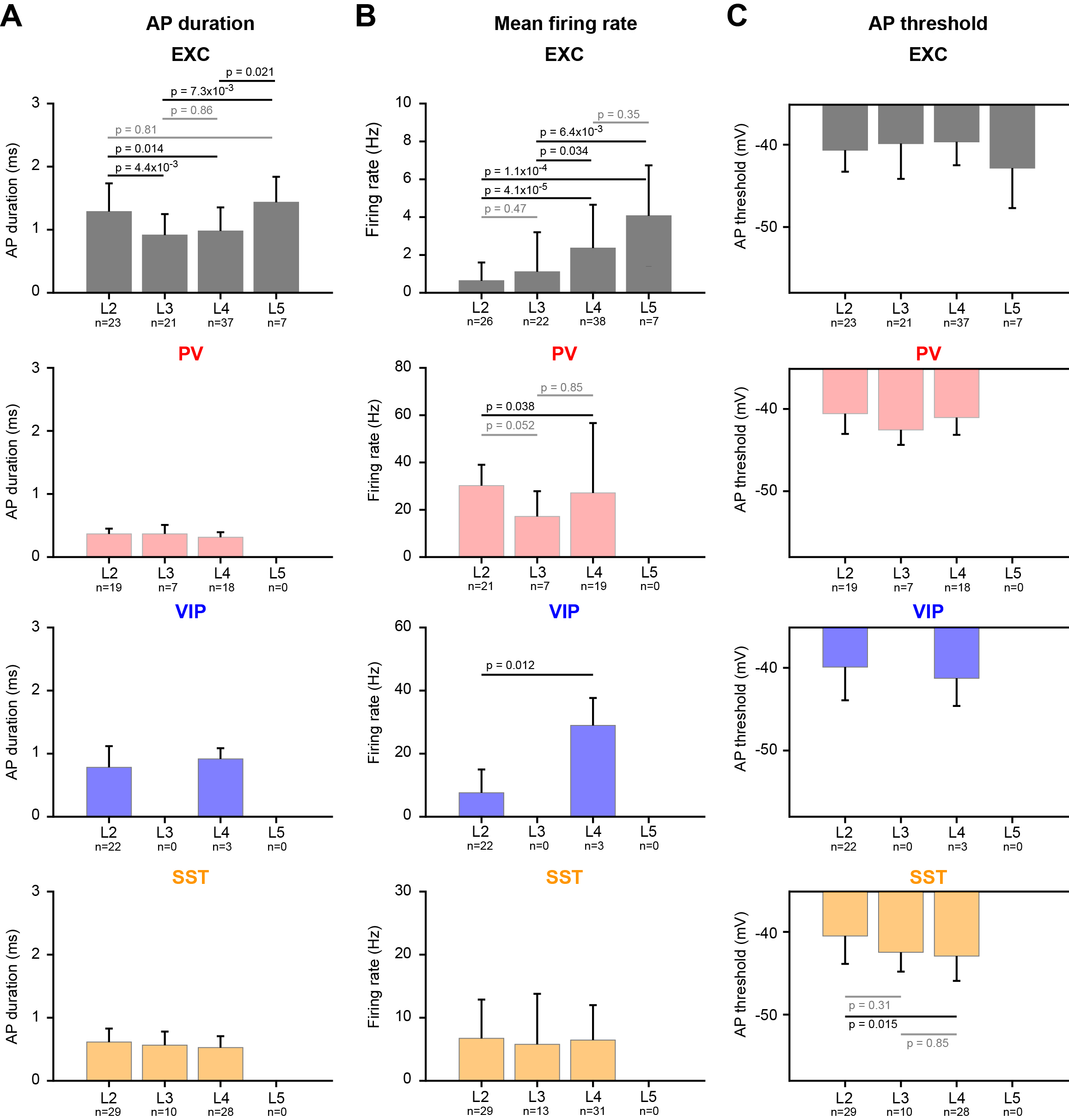

Supplement: S2 Fig — Related to Fig 2. (A) Mean action potential (AP) duration for epochs of quiet wakefulness across cortical layers for each cell class. Bars and error bars represent mean and SD, respectively. The number of cells in each layer is indicated below each bar. Statistical differences between layers were tested using a Kruskal-Wallis test (EXC, p = 1.8x10^-4^; PV, p = 0.29; VIP, p = 0.18; SST, p = 0.12) followed by a Tukey-Kramer multiple comparison test, when appropriate (p values indicated on the graph in grey or black for non-significant and significant differences, respectively). (B) Same as A, but for the mean AP firing rate (Kruskal-Wallis: EXC, p = 9.0x10^-7^; PV, p = 0.014; VIP, p = 0.012; SST, p = 0.55). (C) Same as A, but for the mean AP threshold (Kruskal-Wallis: EXC, p = 0.17; PV, p = 0.13; VIP, p = 0.62; SST, p = 0.018). (JPG) [file pone.0287174.s006.jpg]

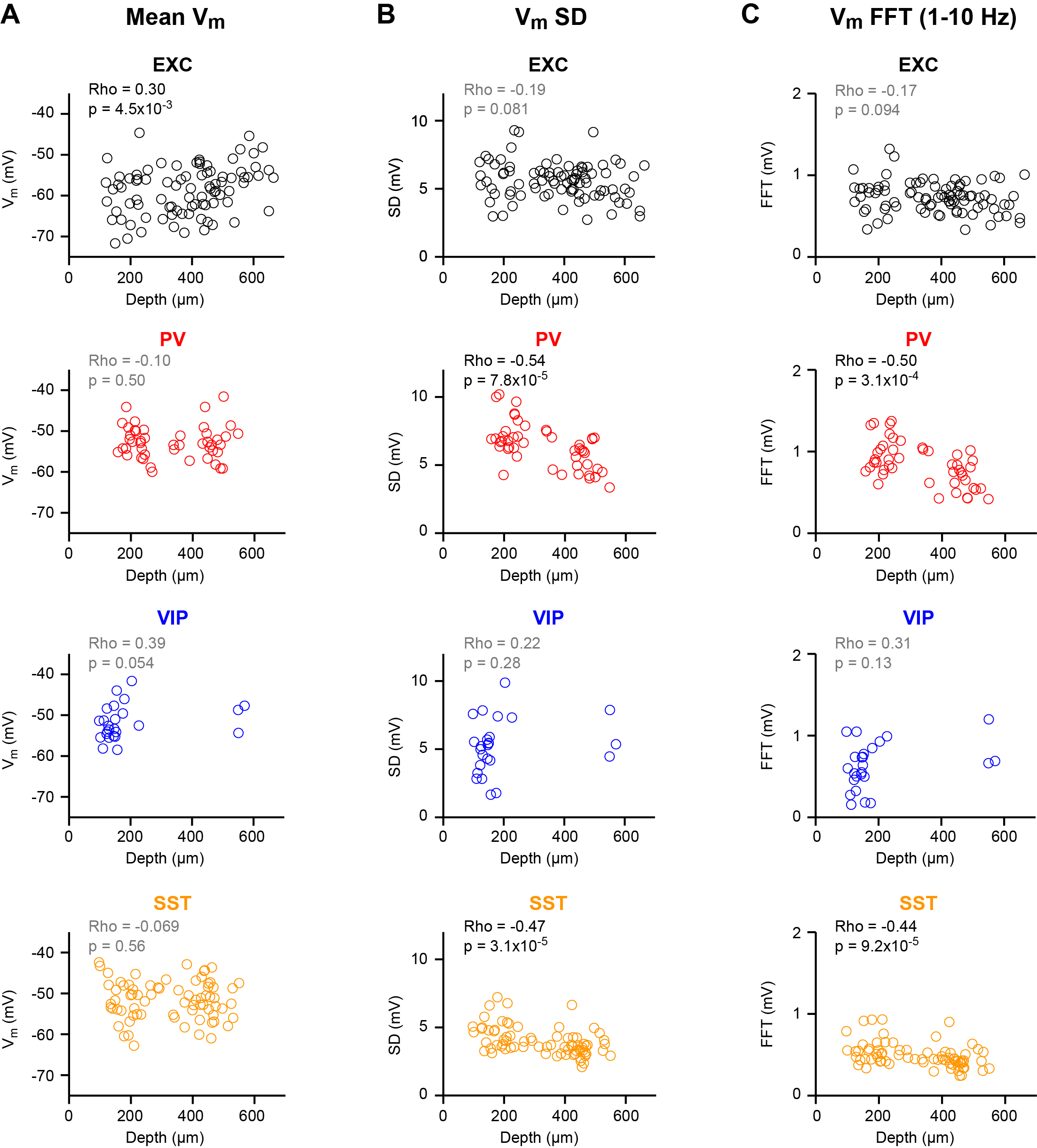

Supplement: S3 Fig — Related to Fig 2. (A) Mean membrane potential (Vm) for epochs of quiet wakefulness across cell depth for each cell class. Open circles represent single neurons. Correlation between mean Vm and cell depth was assessed using Spearman test; Spearman correlation coefficient (Rho) and p value are indicated on each graph. (B) Same as A, but for the mean standard deviation (SD) of the Vm. (C) Same as A, but for the mean 1–10 Hz Vm FFT amplitude. (JPG) [file pone.0287174.s007.jpg]

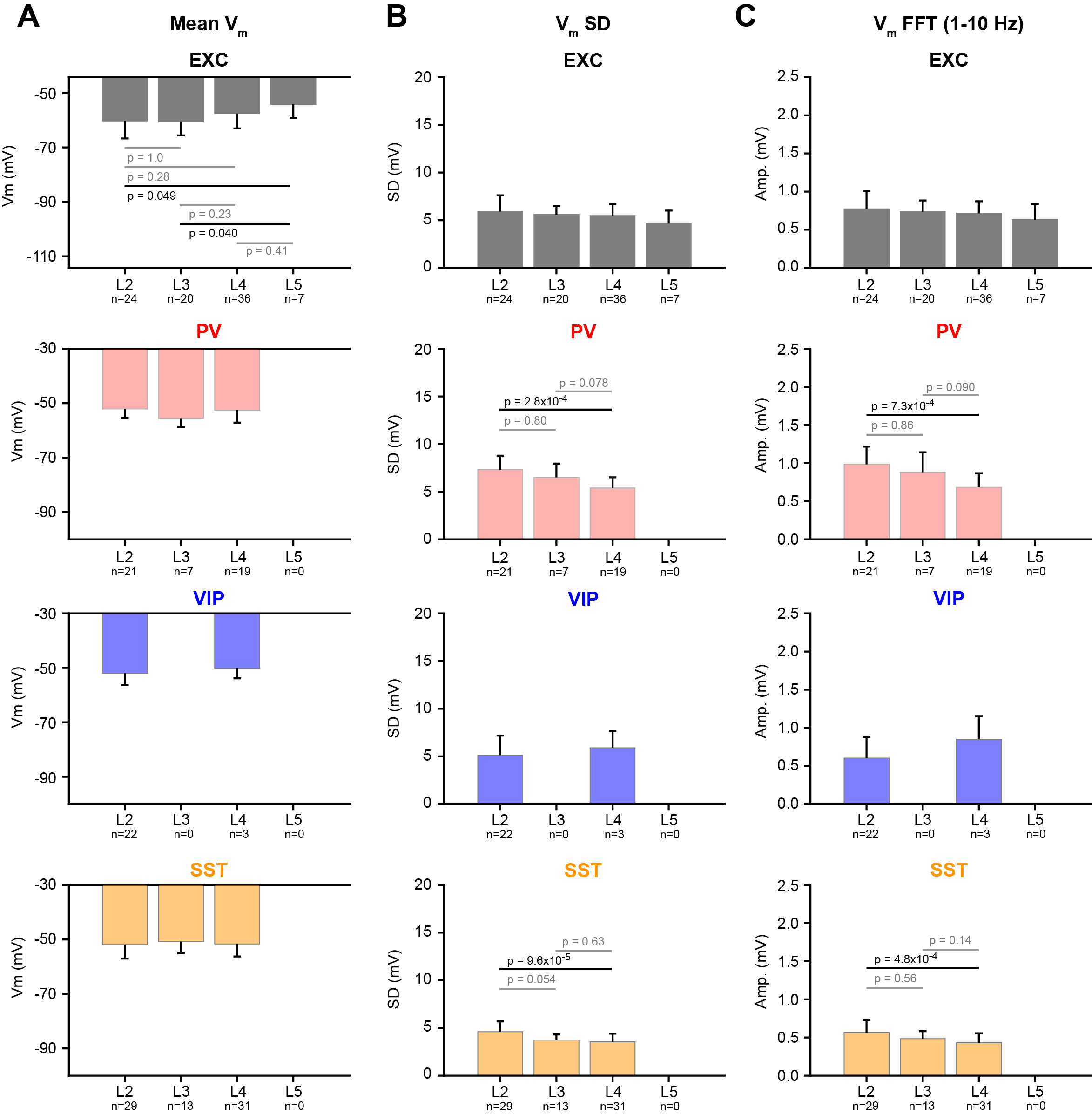

Supplement: S4 Fig — Related to Fig 2. (A) Mean membrane potential (Vm) for epochs of quiet wakefulness across cortical layers for each cell class. Bars and error bars represent mean and SD, respectively. The number of cells in each layer is indicated below each bar. Statistical differences between layers were tested using a Kruskal-Wallis test (EXC, p = 0.016; PV, p = 0.11; VIP, p = 0.40; SST, p = 0.83) followed by a Tukey-Kramer multiple comparison test, when appropriate (p values indicated on the graph in grey or black for non-significant and significant differences, respectively). (B) Same as A, but for the mean standard deviation (SD) of the Vm (Kruskal-Wallis: EXC, p = 0.23; PV, p = 3.7x10^-4^; VIP, p = 0.50; SST, p = 1.4x10^-4^). (C) Same as A, but for the mean 1–10 Hz Vm FFT amplitude (Kruskal-Wallis: EXC, p = 0.34; PV, p = 9.0x10^-4^; VIP, p = 0.21; SST, p = 7.2x10^-4^). (JPG) [file pone.0287174.s008.jpg]

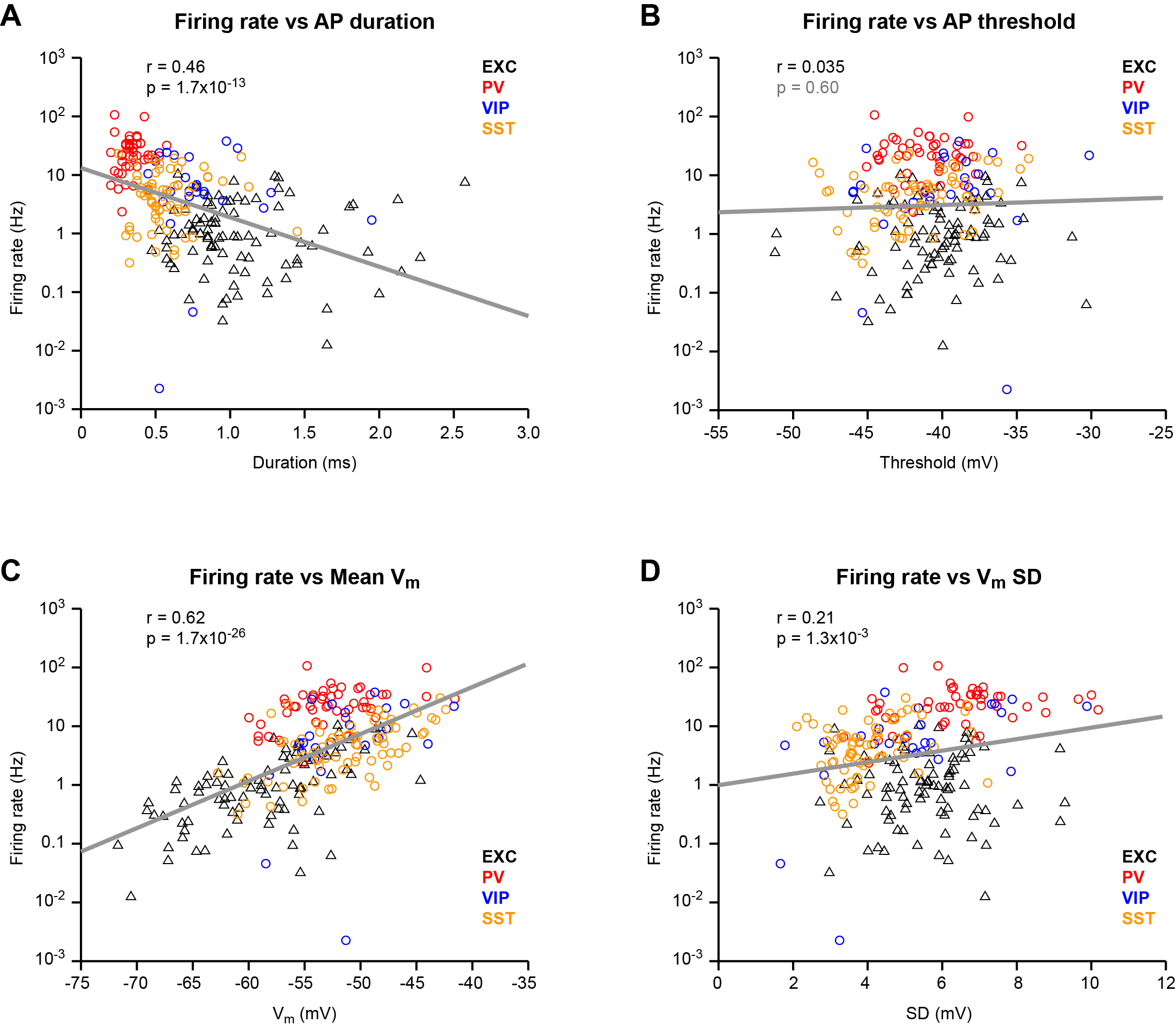

Supplement: S5 Fig — Related to Fig 2. (A) Mean firing rate (logarithmic scale) vs mean AP duration. Open triangles represent single excitatory neurons. Open circles represent inhibitory neurons expressing PV (red), VIP (blue) or SST (orange). Correlation between Log10(firing rate) and AP duration was assessed using a Pearson test. (B) Same as A, but for the mean firing rate vs AP threshold. (C) Same as A, but for the mean firing rate vs mean Vm. (D) Same as A, but for the mean firing rate vs mean standard deviation (SD) of the Vm. (JPG) [file pone.0287174.s009.jpg]

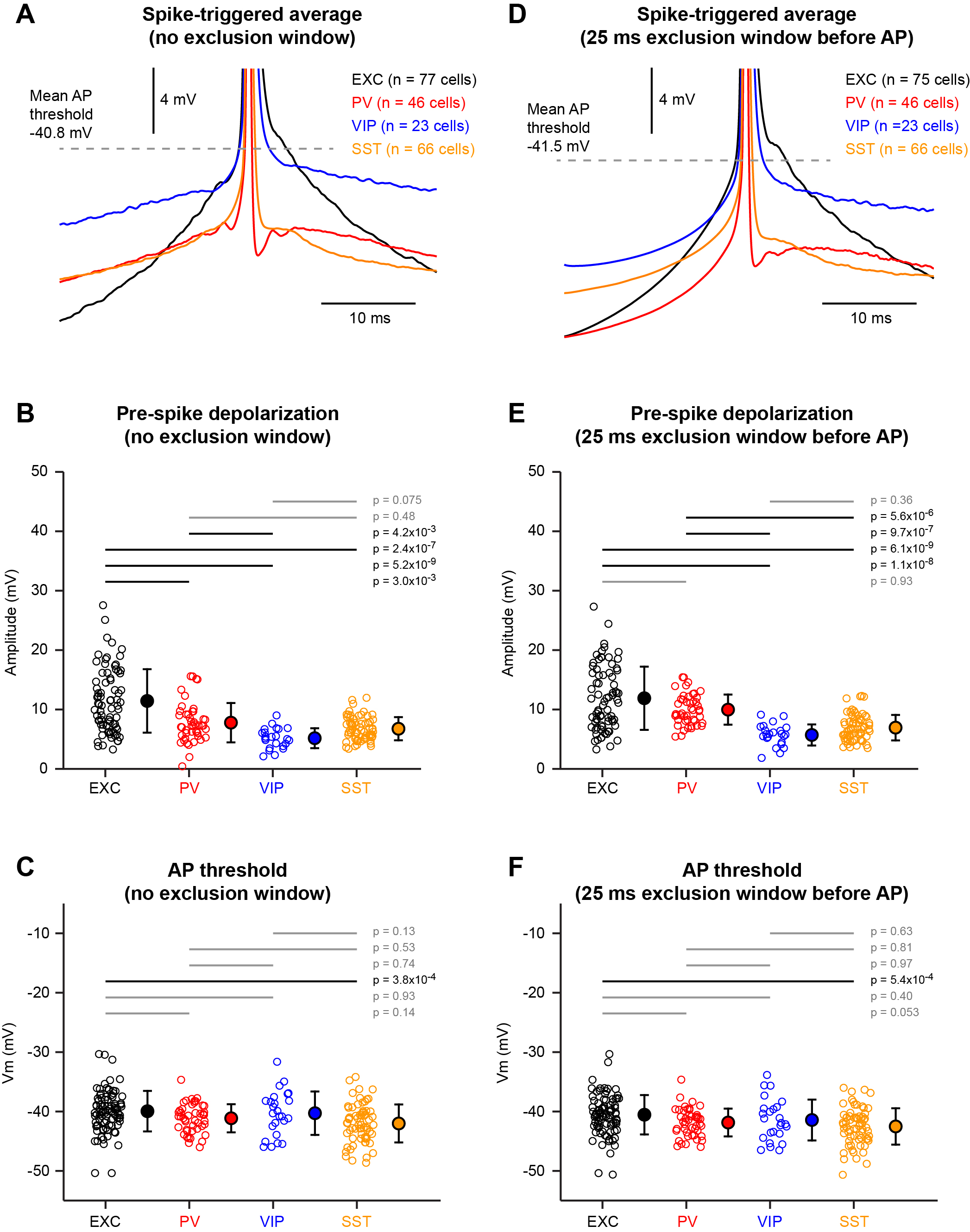

Supplement: S6 Fig — Related to Fig 2. (A) Grand-average spike-triggered Vm for excitatory (EXC, black) and inhibitory neurons expressing PV (red), VIP (blue) or SST (orange). The grey dashed-line indicates the mean AP threshold across all cell classes. (B) Depolarization before AP initiation computed as the change in Vm 20 ms before AP initiation relative to AP threshold. Open circles show individual neuron values. Filled circles with error bars show mean ± SD. Statistical differences between cell classes were computed using a Kruskal-Wallis test (p = 1.5x10^-11^) followed by a Tukey-Kramer multiple comparison test. (C) Mean AP threshold. Open circles show individual neuron values. Filled circles with error bars show mean ± SD. Statistical differences between cell classes were computed using a Kruskal-Wallis test (p = 7.2x10^-4^) followed by a Tukey-Kramer multiple comparison test. (D) Same as A, but computed only for all APs not preceded by another AP within 25 ms. (E) Same as B, but computed for APs with 25 ms exclusion window before AP (Kruskal-Wallis test, p = 2.1x10^-14^). (F) Same as C, but computed for APs with 25 ms exclusion window before AP (Kruskal-Wallis test, p = 8.7x10^-4^). (JPG) [file pone.0287174.s010.jpg]

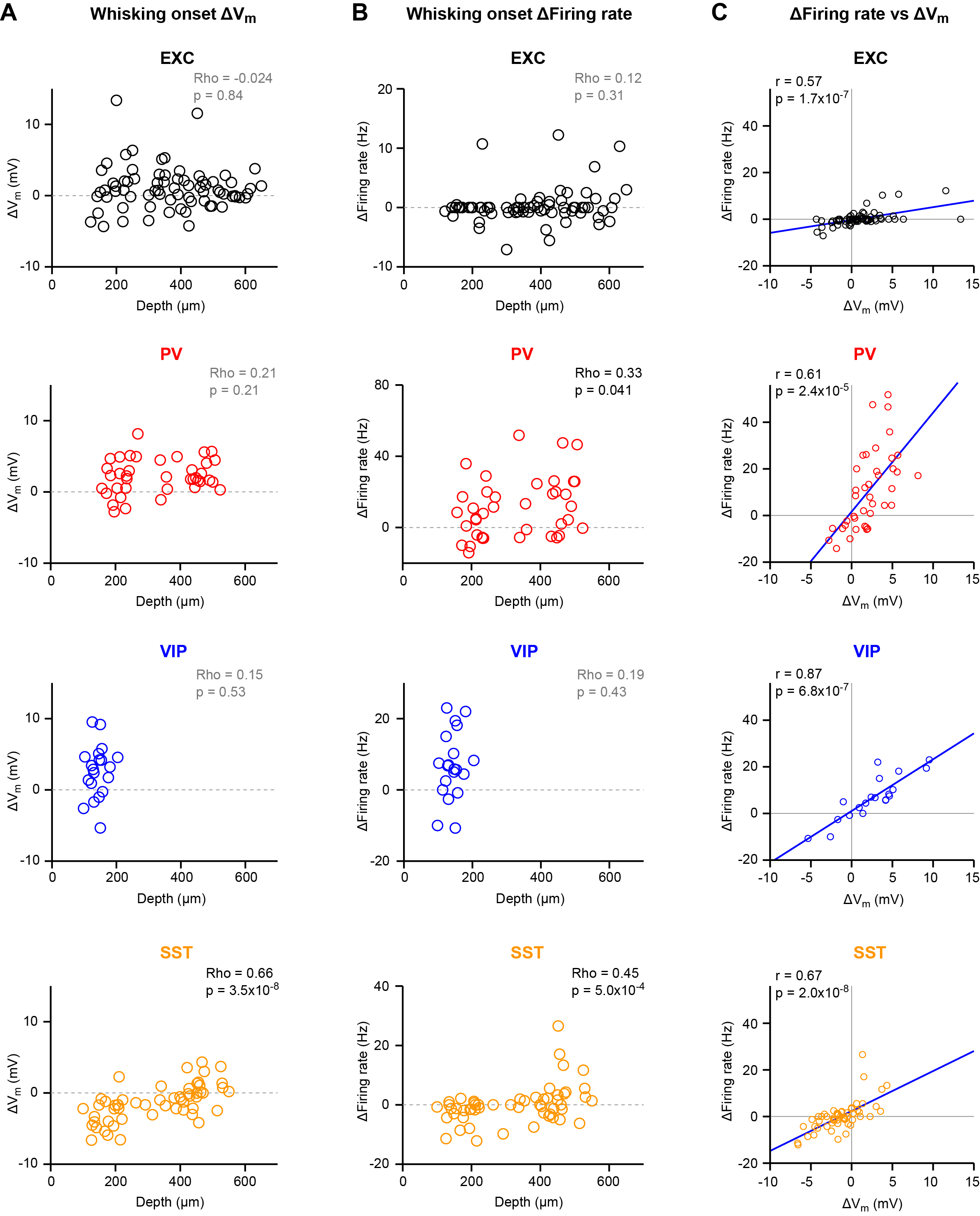

Supplement: S7 Fig — Related to Fig 3. (A) Change in Vm at whisking onset across cell depth for each cell class. Open circles represent single neurons. Correlation between Vm change and cell depth was assessed using a Spearman test; Spearman correlation coefficient (Rho) and p value are indicated on each graph. (B) Same as A, but for the change in firing rate. (C) Change in firing rate vs change in Vm at whisking onset for the four cell classes. Correlation between the change in firing rate and the change in Vm was assessed using a Pearson test; Pearson correlation coefficient (r) and p value are indicated on each graph. (JPG) [file pone.0287174.s011.jpg]

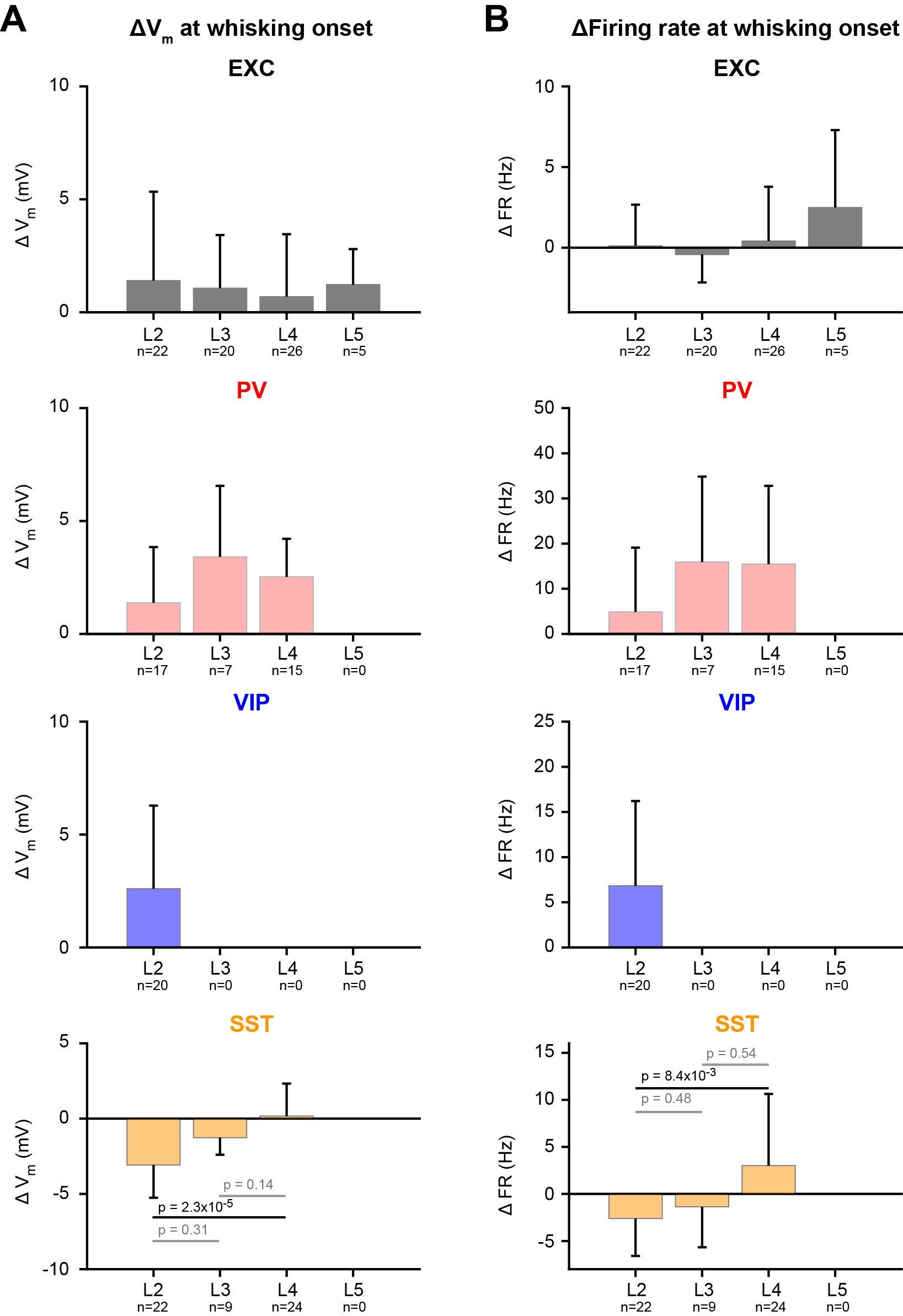

Supplement: S8 Fig — Related to Fig 3. (A) Change in Vm at whisking onset across cortical layers for each cell class. Bars and error bars represent mean and SD, respectively. The number of cells in each layer is indicated below each bar. Statistical differences between layers were tested using a Kruskal-Wallis test (EXC, p = 0.74; PV, p = 0.30; SST, p = 4.5x10^-5^) followed by a Tukey-Kramer multiple comparison test, when appropriate (p values indicated on the graph in grey or black for non-significant and significant differences, respectively). (B) Same as A, but for the change in firing rate (Kruskal-Wallis: EXC, p = 0.61; PV, p = 0.14; SST, p = 0.012). (JPG) [file pone.0287174.s012.jpg]

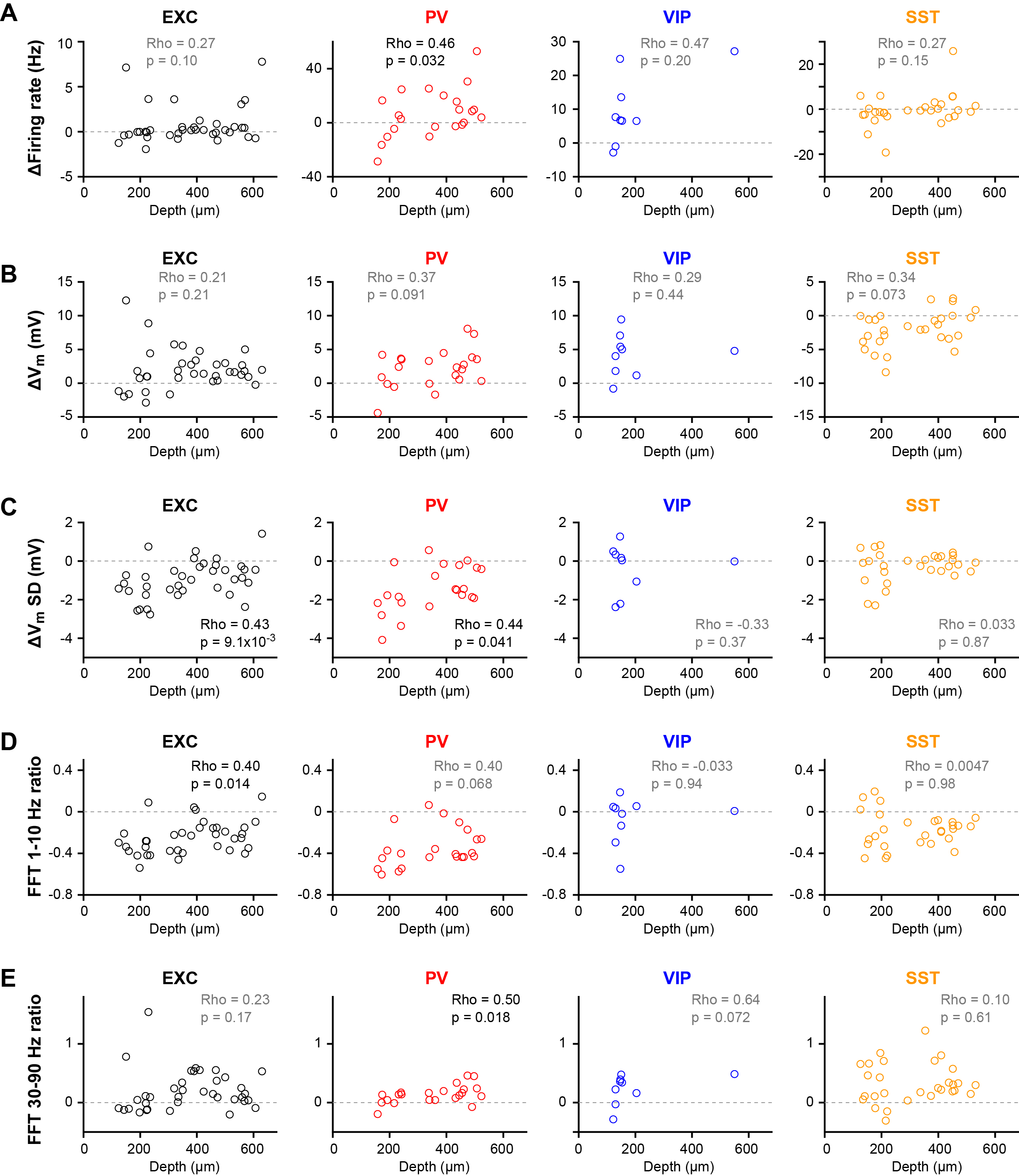

Supplement: S9 Fig — Related to Fig 4. (A) Difference in firing rate (whisking minus quiet wakefulness) across cell depth for each cell class. Open circles represent single neurons. Correlation between difference in firing rate and cell depth was assessed using a Spearman test; Spearman correlation coefficient (Rho) and p value are indicated on each graph. (B) Same as A, but for the difference in mean Vm. (C) Same as A, but for the difference in mean standard deviation (SD) of the Vm. (D) Same as A, but for the relative change in 1–10 Hz Vm FFT amplitude (whisking minus quiet wakefulness divided by quiet wakefulness). (E) Same as A, but for the relative change in 30–90 Hz Vm FFT amplitude. (JPG) [file pone.0287174.s013.jpg]

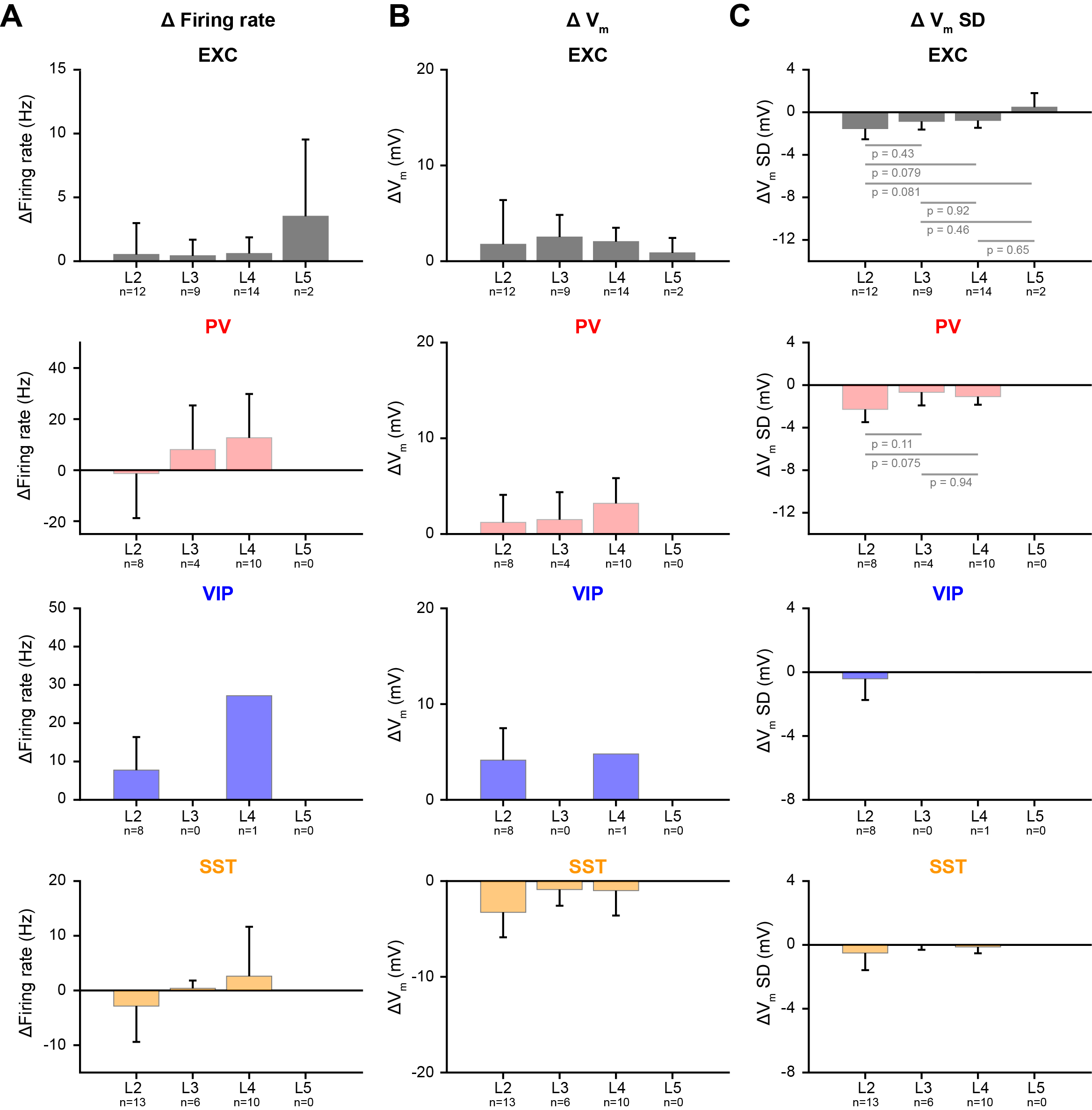

Supplement: S10 Fig — Related to Fig 4. (A) Difference in firing rate (whisking minus quiet wakefulness) across cortical layers for each cell class. Bars and error bars represent mean and SD, respectively. The number of cells in each layer is indicated below each bar. Statistical differences between layers were tested using a Kruskal-Wallis test (EXC, p = 0.55; PV, p = 0.27; SST, p = 0.15) followed by a Tukey-Kramer multiple comparison test, when appropriate (p values indicated on the graph in grey or black for non-significant and significant differences, respectively). (B) Same as A, but for the difference in mean Vm (Kruskal-Wallis test, EXC, p = 0.36; PV, p = 0.49; SST, p = 0.090). (C) Same as A, but for the difference in mean standard deviation (SD) of the Vm (Kruskal-Wallis test, EXC, p = 0.030; PV, p = 0.046; SST, p = 0.65). (JPG) [file pone.0287174.s014.jpg]

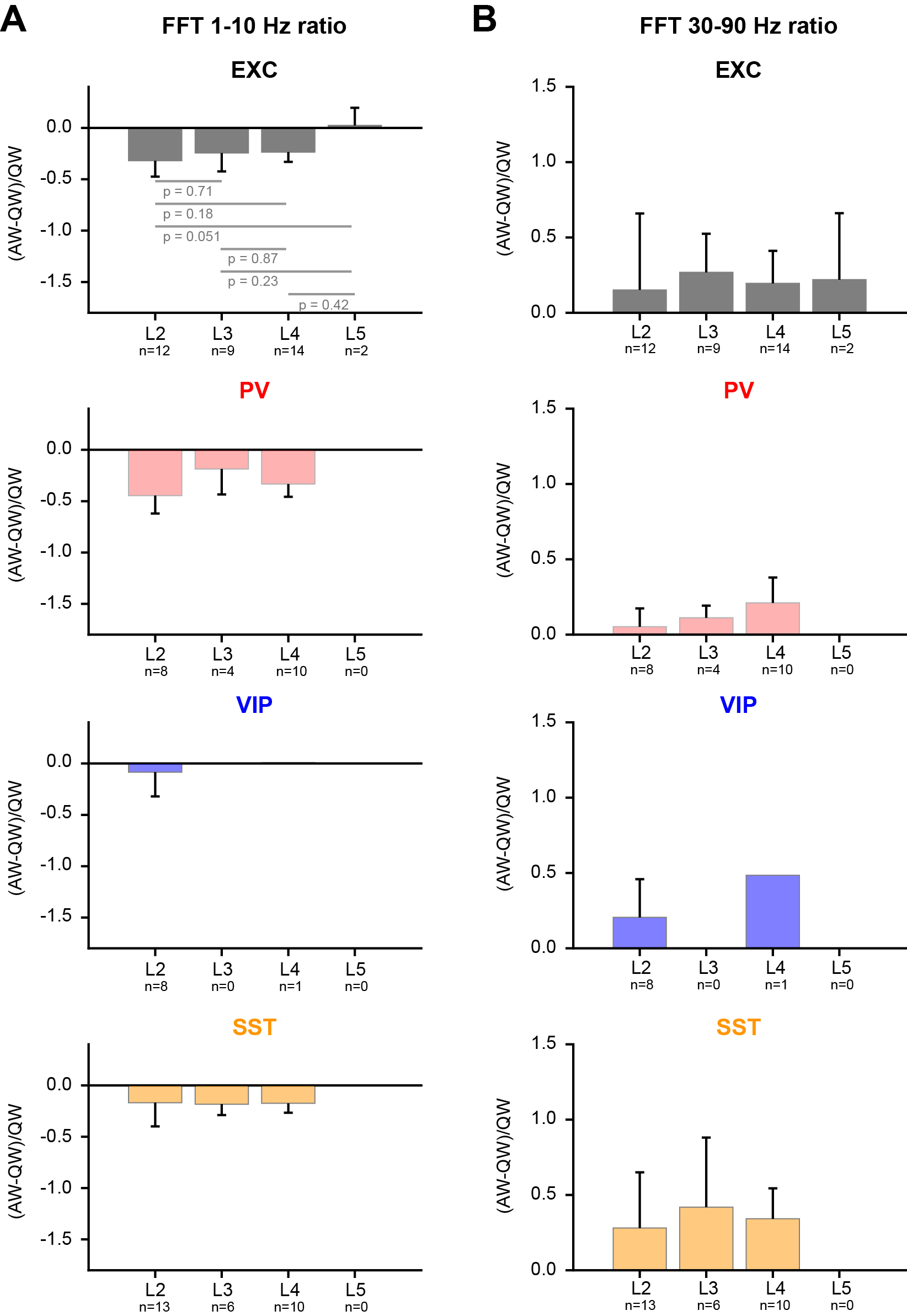

Supplement: S11 Fig — Related to Fig 4. (A) Relative change in mean 1–10 Hz FFT amplitude (whisking minus quiet wakefulness divided by quiet wakefulness) across cortical layers for each cell class. Bars and error bars represent mean and SD, respectively. The number of cells in each layer is indicated below each bar. Statistical differences between layers were tested using a Kruskal-Wallis test (EXC, p = 0.037; PV, p = 0.075; SST, p = 0.94) followed by a Tukey-Kramer multiple comparison test, when appropriate (p values indicated on the graph in grey or black for non-significant and significant differences, respectively). (B) Same as A, but for the difference in 30–90 Hz Vm FFT amplitude (Kruskal-Wallis test: EXC, p = 0.24; PV, p = 0.13; SST, p = 0.66). (JPG) [file pone.0287174.s015.jpg]

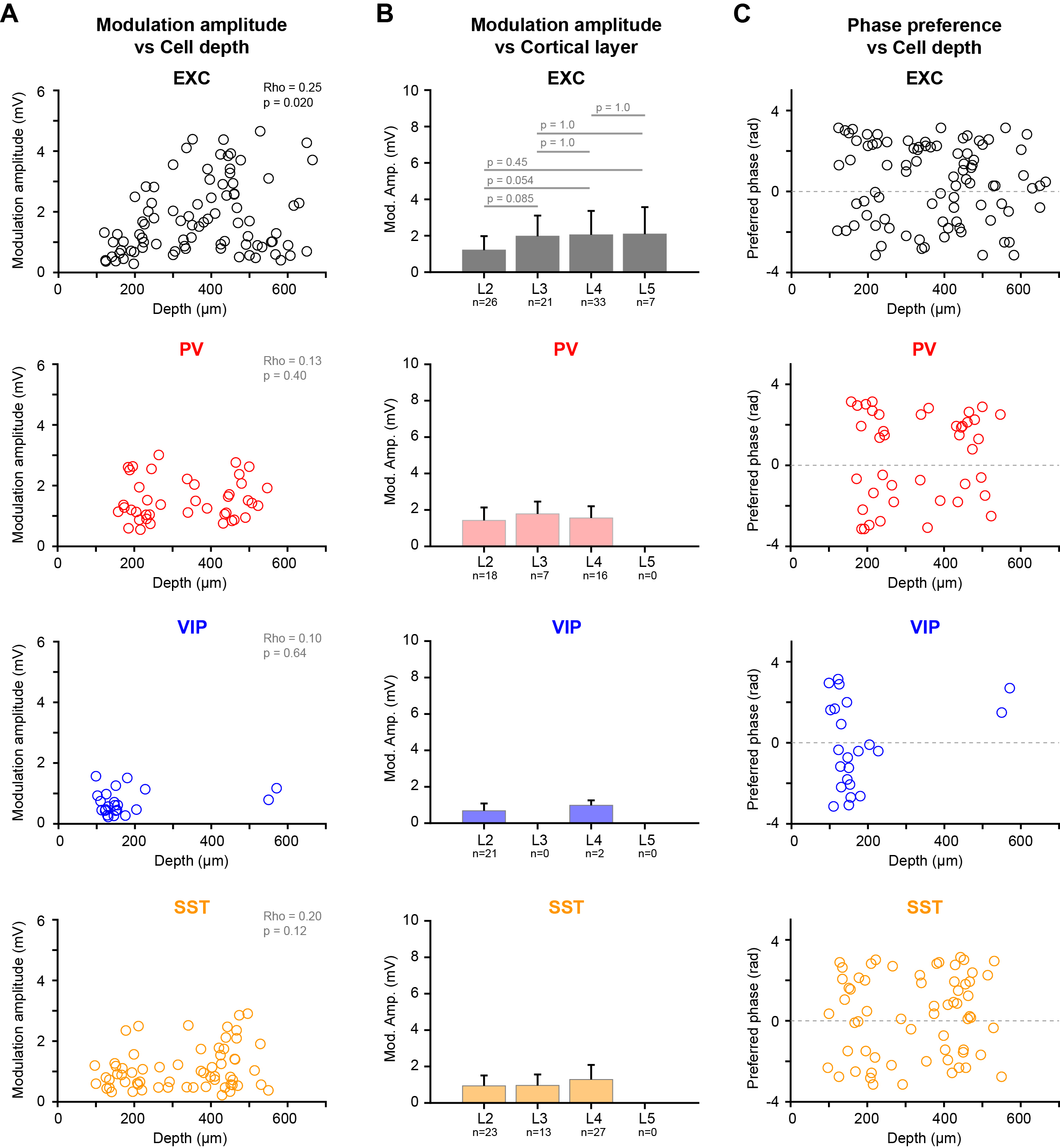

Supplement: S12 Fig — Related to Fig 5. (A) Mean Vm phase modulation amplitude across cell depth for each cell class. Open circles represent single neurons. Correlation between the modulation amplitude and cell depth was assessed using a Spearman test; Spearman correlation coefficient (Rho) and p value are indicated on each graph. (B) Mean Vm phase modulation amplitude across cortical layers for each cell class. Bars and error bars represent mean and SD, respectively. The number of cells in each layer is indicated below each bar. Statistical differences between layers were tested using a Kruskal-Wallis test (EXC, p = 0.041; PV, p = 0.35; VIP, p = 0.19; SST, p = 0.30) followed by a Tukey-Kramer multiple comparison test, when appropriate (p values indicated on the graph in grey or black for non-significant and significant differences, respectively). (C) Same as A, but for the phase preference. (JPG) [file pone.0287174.s016.jpg]

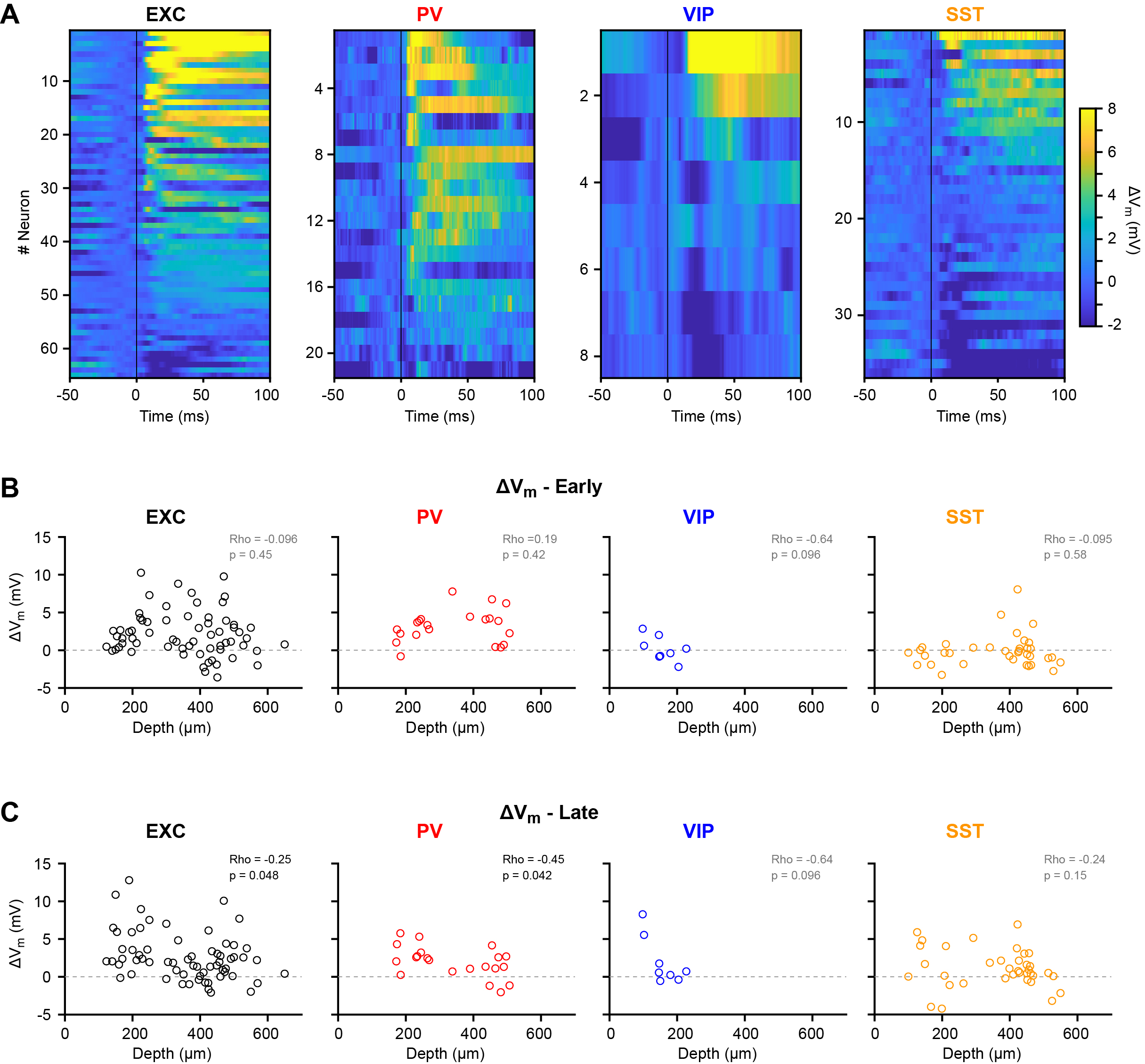

Supplement: S13 Fig — Related to Fig 6. (A) Color-coded average single-neuron Vm responses to active touch onset for each cell class. Neurons are sorted by the amplitude of the peak response. (B) Change in Vm in the early (5–20 ms) time window after touch onset across cell depth for each cell class. Open circles represent single neurons. Correlation between Vm change and cell depth was assessed using a Spearman test; Spearman correlation coefficient (Rho) and p value are indicated on each graph. (C) Same as B, but for the late (30–100 ms) time window after touch onset. (JPG) [file pone.0287174.s017.jpg]

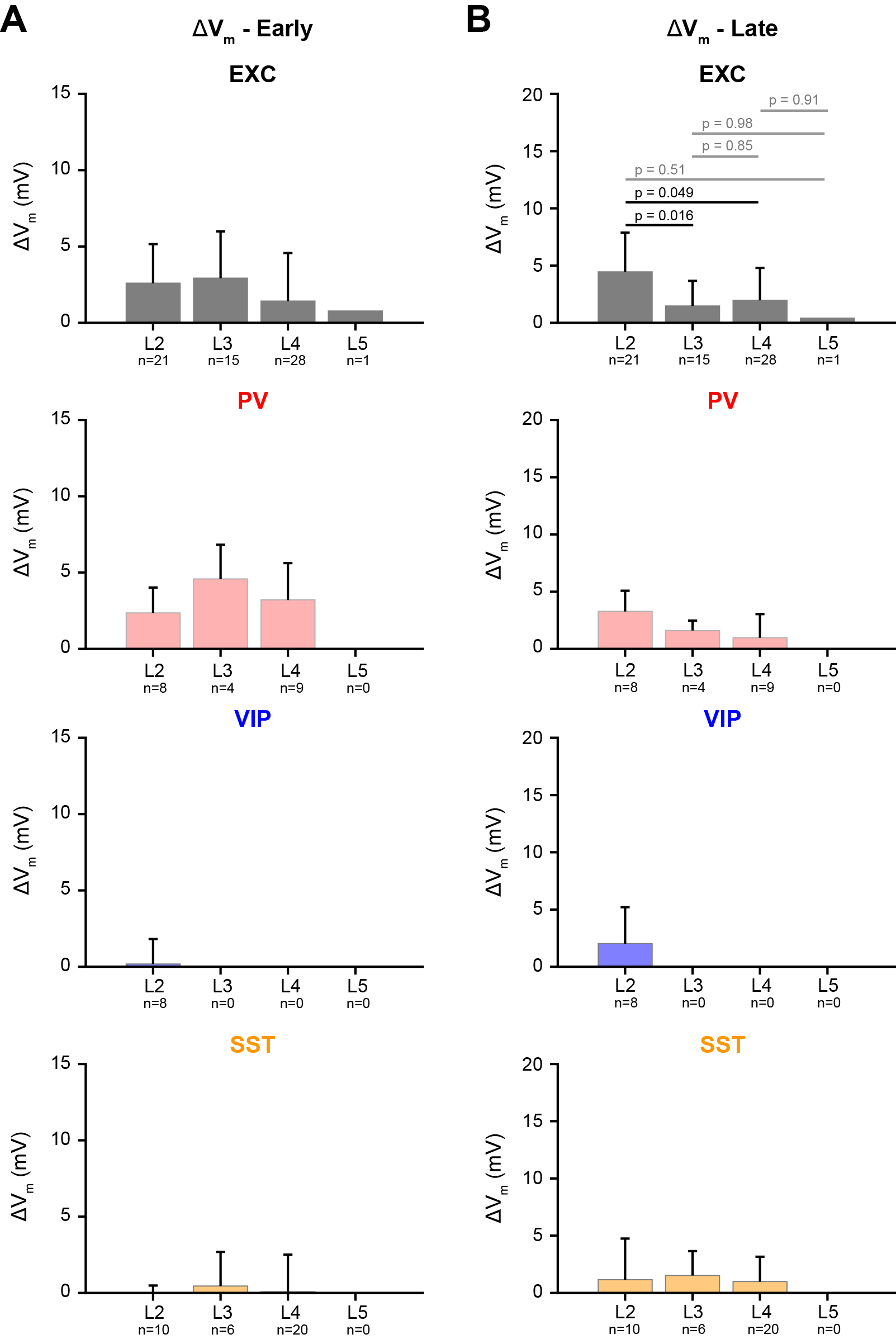

Supplement: S14 Fig — Related to Fig 6. (A) Change in Vm in the early (5–20 ms) time window after touch onset across cortical layers for each cell class. Bars and error bars represent mean and SD, respectively. The number of cells in each layer is indicated below each bar. Statistical differences between layers were tested using a Kruskal-Wallis test (EXC, p = 0.38; PV, p = 0.26; SST, p = 0.58) followed by a Tukey-Kramer multiple comparison test, when appropriate (p values indicated on the graph in grey or black for non-significant and significant differences, respectively). (B) Same as B, but for the late (30–100 ms) time window after touch onset (Kruskal-Wallis test: EXC, p = 0.011; PV, p = 0.058; SST, p = 0.89). (JPG) [file pone.0287174.s018.jpg]

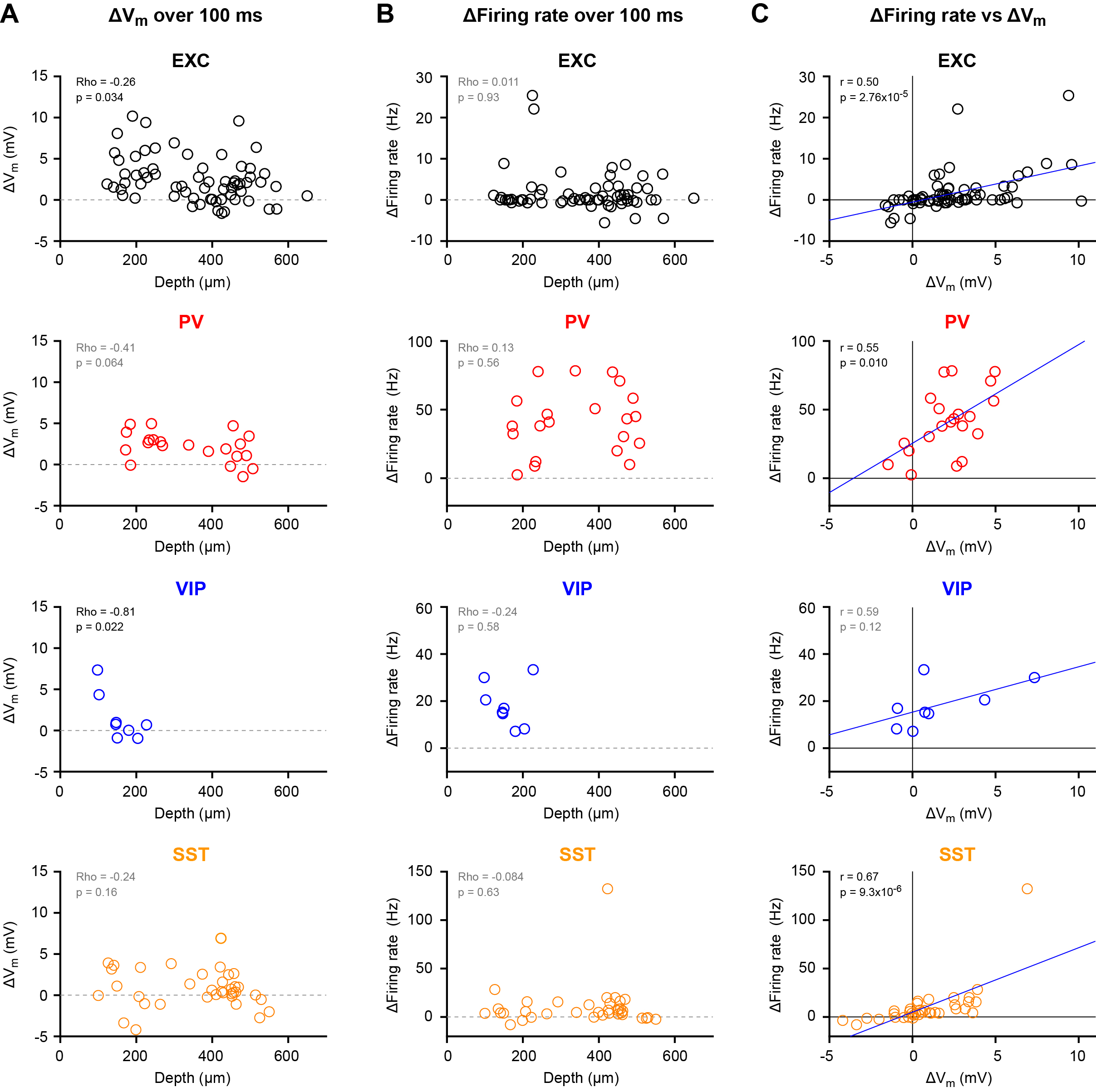

Supplement: S15 Fig — Related to Fig 6. (A) Change in Vm (0–100 ms) after touch onset across cell depth for each cell class. Open circles represent single neurons. Correlation between Vm change and cell depth was assessed using a Spearman test; Spearman correlation coefficient (Rho) and p value are indicated on each graph. (B) Same as A, but for the change in firing rate. (C) Change in firing rate vs change in Vm. The correlation was assessed using a Pearson test; Pearson correlation coefficient (r) and p value are indicated on each graph. (JPG) [file pone.0287174.s019.jpg]

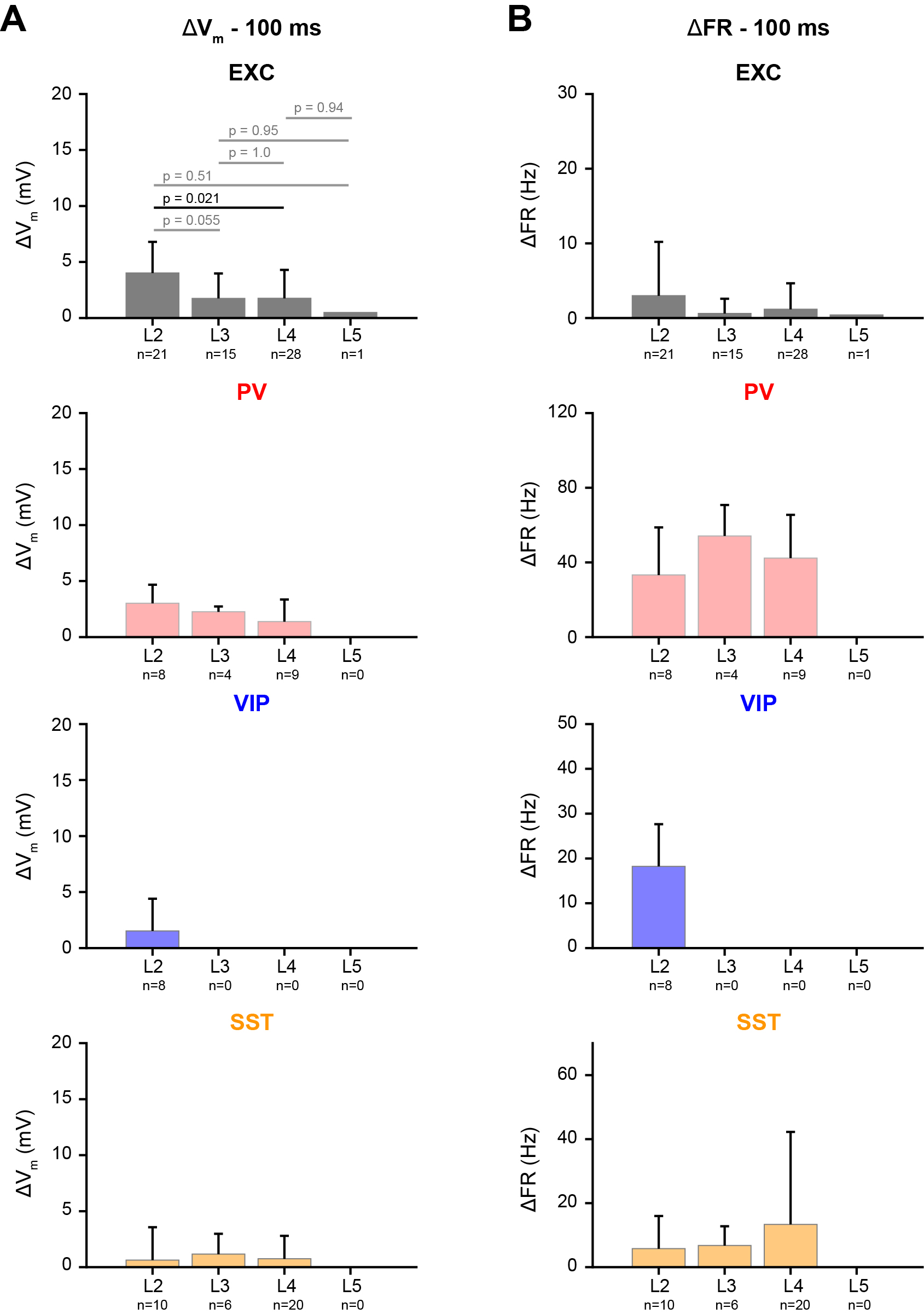

Supplement: S16 Fig — Related to Fig 6. (A) Change in Vm (0–100 ms) after touch onset across cortical layers for each cell class. Bars and error bars represent mean and SD, respectively. The number of cells in each layer is indicated below each bar. Statistical differences between layers were tested using a Kruskal-Wallis test (EXC, p = 0.014; PV, p = 0.12; SST, p = 0.91) followed by a Tukey-Kramer multiple comparison test, when appropriate (p values indicated on the graph in grey or black for non-significant and significant differences, respectively). (B) Same as A, but for the change in firing rate (Kruskal-Wallis: EXC, p = 0.85; PV, p = 0.23; SST, p = 0.75). (JPG) [file pone.0287174.s020.jpg]

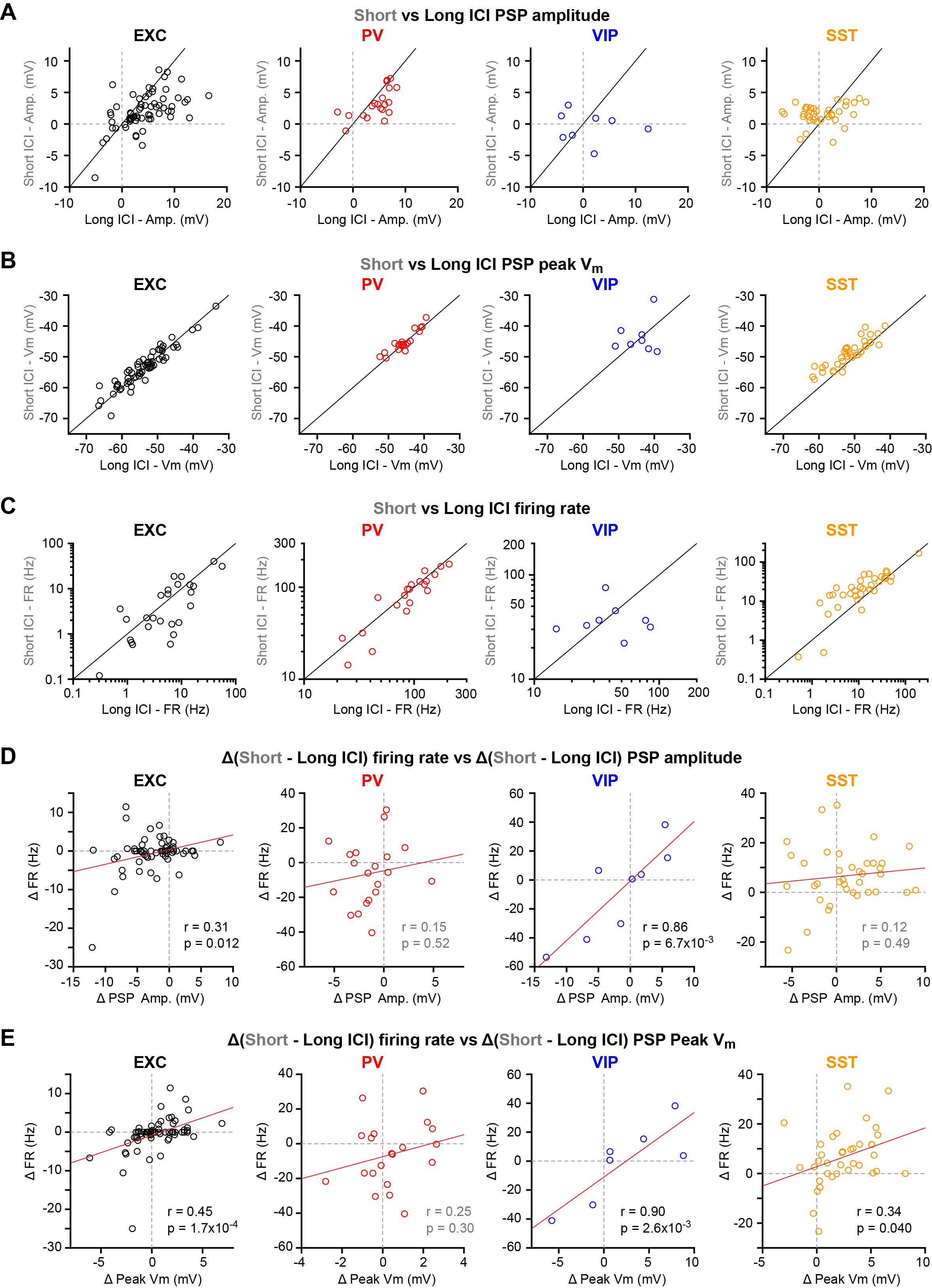

Supplement: S17 Fig — Related to Fig 7. (A) Postsynaptic potential (PSP) amplitude for short vs long intercontact intervals for each cell class. Open circles represent single neurons. (B) Same as A, but for peak PSP Vm. (C) Same as A, but for firing rate. (D) Difference in firing rate vs difference in PSP amplitude between short and long intercontact intervals. Correlation was assessed using a Pearson test; Pearson correlation coefficient (r) and p value are indicated on each graph. (E) Same as D, but for the difference in firing rate vs the difference in peak PSP Vm. (JPG) [file pone.0287174.s021.jpg]

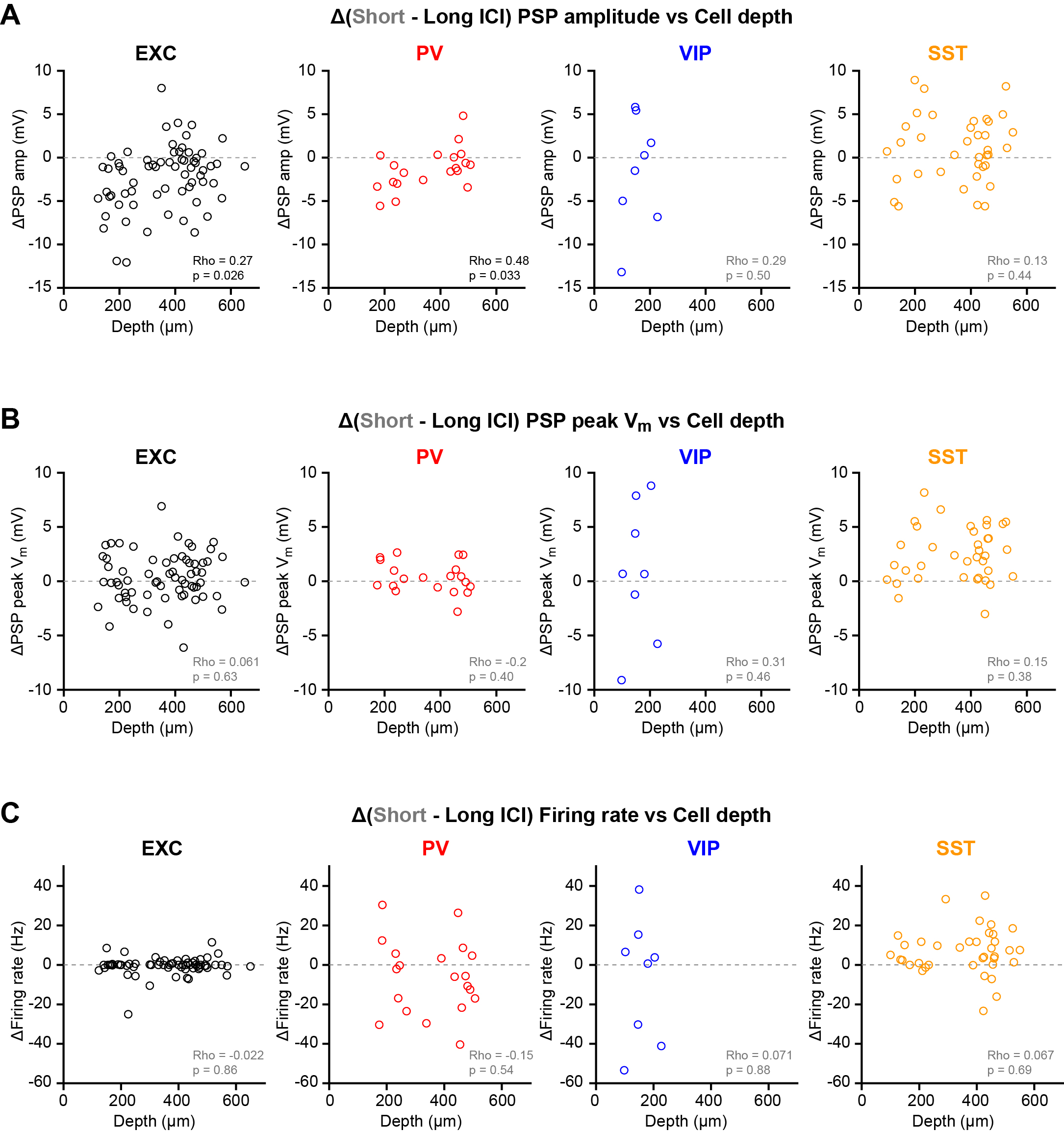

Supplement: S18 Fig — Related to Fig 7. (A) Mean difference in postsynaptic potential (PSP) amplitude between short and long intercontact intervals across cell depth for each cell class. Open circles represent single neurons. Correlation between the difference in PSP amplitude and cell depth was assessed using a Spearman test; Spearman correlation coefficient (Rho) and p value are indicated on each graph. (B) Same as A, but for the difference in Vm at the peak of the PSP. (C) Same as A, but for the difference in firing rate. (JPG) [file pone.0287174.s022.jpg]

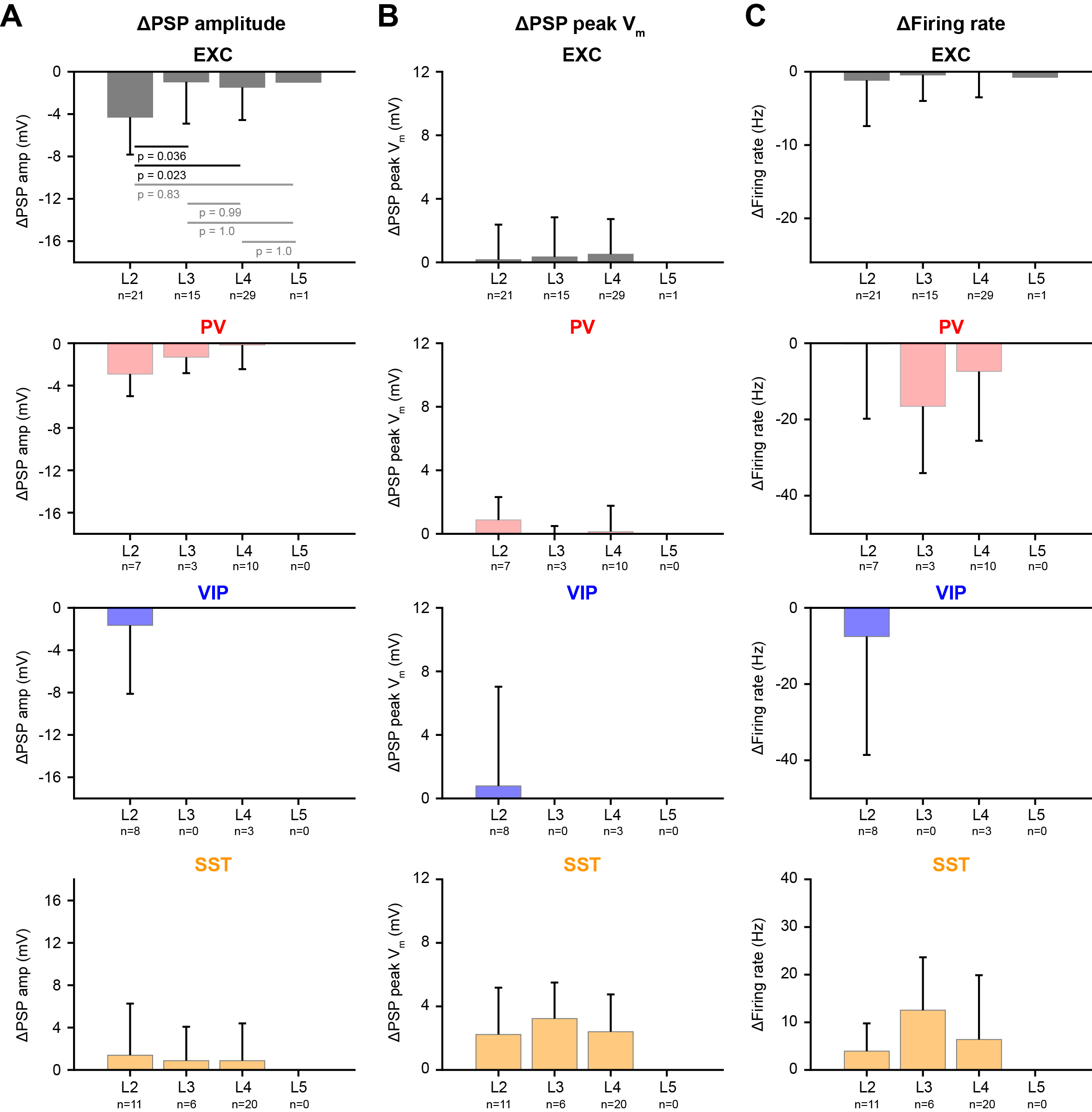

Supplement: S19 Fig — Related to Fig 7. (A) Mean difference in postsynaptic potential (PSP) amplitude between short and long intercontact intervals across cortical layers for each cell class. Bars and error bars represent mean and SD, respectively. The number of cells in each layer is indicated below each bar. Statistical differences between layers were tested using a Kruskal-Wallis test (EXC, p = 0.015; PV, p = 0.075; SST, p = 0.97) followed by a Tukey-Kramer multiple comparison test when appropriate (p values indicated on the graph in grey or black for non-significant and significant differences, respectively). (B) Same as A, but for the difference in Vm at the peak of the PSP (Kruskal-Wallis test: EXC, p = 0.94; PV, p = 0.59; SST, p = 0.59). (C) Same as A, but for the difference in firing rate (Kruskal-Wallis test: EXC, p = 0.58; PV, p = 0.38; SST, p = 0.26). (JPG) [file pone.0287174.s023.jpg]
